# Commentary: Pembrolizumab-induced type-1 diabetes mellitus: clinical characteristics, therapeutic strategies, and prognosis from a retrospective analysis of 43 published cases

**DOI:** 10.3389/fendo.2026.1986405

**Published:** 2026-09-29

**Authors:** Shinichiro M. Koga

**Affiliations:** Department of Diabetes, Endocrinology and Metabolism, Tokyo Metropolitan Center for Cancer and Infectious Diseases, Komagome Hospital, Tokyo, Japan

**Keywords:** diabetic ketoacidosis, hyperglycemia, immune checkpoint inhibitor, immune checkpoint inhibitor-associated diabetes, immune-related adverse event, insulin therapy, pembrolizumab, type 1 diabetes

## Introduction

Guo et al. provide an important characterization of pembrolizumab-induced T1DM based on 43 published cases (1). Diabetic ketoacidosis occurred in 36 patients (83.7%), C-peptide was low in 30 of 34 evaluable patients (88.2%), and 32 of 33 patients with follow-up had persistent insulin dependence or β-cell deficiency (1). Islet autoantibodies were detected in only 38.5%; among the reported tests, anti-GAD antibodies were positive in 12 of 39 patients (30.8%), anti-IA-2 antibodies in 3 of 20 (15.0%), islet cell antibodies (ICA) in 2 of 14 (14.3%), and insulin autoantibodies (IAA) in 1 of 16 (6.3%) (1). Thus, negative autoantibody results do not exclude the diagnosis (1). These findings establish the severity of clinically apparent ICI-associated T1DM. They also raise a clinically important question: must clinicians wait until overt insulin deficiency or DKA has developed before initiating insulin?

## The unresolved therapeutic window

Guo et al. appropriately emphasize glucose monitoring and prompt assessment of hyperglycemic symptoms (1). As well as they have characterized what happens when ICI-associated diabetes becomes clinically overt, but their dataset cannot determine when treatment should begin during the preceding hyperglycemic phase.

This distinction is important. Once clinically apparent ICI-associated T1DM develops, endogenous insulin secretion is usually profoundly impaired and recovery is uncommon (1). Thus, the clinical objective of earlier insulin intervention should not be framed as preservation or regeneration of β-cell function. Whether early insulin can alter the underlying β-cell destruction remains unknown.

Rather, the immediate rationale is simpler: prevent severe hyperglycemia, ketosis, and DKA while the diagnostic evaluation is still underway.

## ADA 2026 provides a clinically actionable direction

The Standards of Care in Diabetes—2026 provides an important step toward addressing this gap. Recommendation 9.33 states that individuals who develop hyperglycemia during immunotherapy should be assessed for the immediate need for insulin because of the potential risk of DKA while additional testing is performed to determine whether the hyperglycemia is related to ICI-associated diabetes (2). The accompanying text further states that basal insulin should be considered when blood glucose exceeds 250 mg/dL while evaluation continues (2).

Thus, current guidance does not require complete diagnostic confirmation before insulin is considered. Instead, treatment and diagnostic evaluation can proceed in parallel.

## From early recognition to early insulin intervention

The ADA recommendation provides the clinical framework for considering insulin during diagnostic evaluation, while emerging oncology data illustrate why such an approach may be clinically important. In a 2026 multicenter retrospective study presented at the ASCO Annual Meeting, 27 of 3,254 ICI-treated patients developed autoimmune T1DM (0.83%), and 70% presented with DKA; all patients required insulin (3). These findings support vigilant glucose monitoring and early recognition.

A clinical precedent for intervention before metabolic crisis was reported by Matsuda et al. in a patient receiving durvalumab (4). Basal insulin was initiated when hyperglycemia was present despite preserved endogenous insulin secretion. Although endogenous insulin secretion subsequently declined and ketosis developed, no hyperglycemic crisis occurred during the reported course (4). This case provides a clinical precedent for initiating basal insulin before overt insulin deficiency or DKA, but it does not establish that early insulin changes the underlying β-cell trajectory.

Taken together, these observations suggest a practical clinical principle: When ICI-associated hyperglycemia emerges and appears clinically significant or progressive, insulin should be considered before overt ICI-associated T1DM or DKA develops.

This does not mean that every patient with mild hyperglycemia receiving an ICI should automatically receive insulin. Rather, the decision should incorporate the glucose trajectory, symptoms, ketones, bicarbonate, C-peptide, and rate of deterioration. The ADA 2026 threshold of >250 mg/dL provides a practical point at which basal insulin may be considered while evaluation proceeds (2).

## Discussion

The major contribution of Guo et al. is therefore not only the characterization of pembrolizumab-associated T1DM, but also the identification of a clinical problem that remains unresolved: the optimal point of intervention before overt insulin-deficient diabetes and DKA (1).

We propose that the clinical objective should evolve from simply diagnosing ICI-associated T1DM promptly to identifying clinically significant, evolving ICI-associated insulin deficiency and intervening before metabolic decompensation.

This principle should not be restricted to pembrolizumab or to a particular cancer type. It may be applicable across ICI-treated patients who develop progressive hyperglycemia.

The unresolved question is not whether early insulin preserves β-cell function, but when the risk of metabolic decompensation becomes sufficient to justify insulin before overt ICI-associated diabetes is established.
